# Monastic Jargon and Citizenship Language in Late Antiquity^1^

**DOI:** 10.1080/09503110.2019.1675027

**Published:** 2019-10-31

**Authors:** Claudia Rapp

**Keywords:** Monasticism, Citizenship, Late Antiquity, Christianity, Anthony of Egypt

## Abstract

This article pursues the changing significance associated with the ancient Greek city state (*polis*) in language used among Greek Christian authors of the fourth to sixth centuries CE. In classical Greek writing, the language of the *polis* and related terms (*politeia, politeuma*) play an important role in articulations of the societal contexts for the actions of the individual. Christian authors highlight the communal aspects of belonging to a *polis* to explain the significance of joining the Church through baptism. In the context of early monastic writing, by contrast, it is the personal ascetic achievement of the individual that comes to the fore. The *polis* as a point of reference is transposed to access rights to the Heavenly City that are promised as a reward at the end of time.

## Introduction

**“**Our citizenship (*politeuma*) is in Heaven.” These words in the Letter to the Philippians 3:20 that is attributed to the Apostle Paul invoke the language of city, citizenship and civic conduct to highlight the contrast between the current life with its toils and the life to come with its rewards. The frame of reference in which this passage was employed changed according to the historical circumstances. Once it became clear that the Second Coming of Christ was not imminent, in the course of the first century CE, the eschatological dimension lost its urgency. During the second and third centuries, when Christians were a minority subjected to sporadic persecution, Christian authors, especially apologists, invoked this passage in order to highlight that, while being law-abiding citizens and loyal subjects of the Roman emperor, Christians should live in anticipation of the Heavenly City, often also imagined as the Heavenly Jerusalem.

With the end of the persecutions and the legal recognition of Christianity under the Emperor Constantine in 312 came the growth of the Church in membership and in cultural and political importance. This raised the possibility that the Heavenly City could already be anticipated, in an incomplete and imperfect foreshadowing, in the Church. The expansion of monasticism in the fourth to sixth centuries added a further context for the anticipation of the life of the Heavenly City here on earth, not just in the Church as a whole, but specifically among those who pursued a life dedicated to asceticism. Thus, over time, the prime candidates for citizenship in Heaven were first the martyrs, then the newly baptised Christians and finally the monks.

In the following, I explore the use of citizenship language in the Greek texts generated by the monastic movement in the fourth to sixth centuries CE, expanding and modifying earlier studies of this phenomenon.^2^ It will emerge that, while the use of *polis* language for the Church emphasises the communal aspect, in the monastic context, it is the exercise of appropriate conduct by the individual, through asceticism, that comes to the fore.

## *Polis* language

Expressions centred on the three nouns *polis*, *politeia* and *politeuma* shaped Christian discourse among Greek authors of Late Antiquity, even as the administrative autonomy of the ancient *polis* was being eroded and the urban fabric of many cities was beginning to crumble.^3^ The application of these concepts in the context of religious life, whether Jewish or Christian, has been studied extensively, and helpfully summarised by Michael Hollerich.^4^

Translations of these three terms depend on context and intention. *Polis* has its equivalent in “city”, but with specific resonances to the Greek city states that enjoyed administrative and fiscal autonomy, had their own constitution (*politeia*) and generated a strong sense of civic identity.^5^ *Politeia* designates the constitution of a city, but also the way of life, or conduct, of an individual. *Politeuma* most commonly designates a number of people who are distinguished by sharing the same conduct, but it can also be used as an equivalent to *politeia*, to refer to the conduct itself. Belonging to a *politeuma* often indicates the possession of full membership rights among people who share the same code of conduct.

## Baptismal contexts for *polis* language

Theologians and preachers explained the significance of admission into the Church through baptism by analogy to enrolment in the citizenship list of a *polis*, often with reference to the Letter to the Philippians.

John Chrysostom (d. 407) refers to Paul’s remark that the citizenship (*politeuma*) of Christians is in Heaven (Phil. 3:20) in order to remind his audience of catechumens that they should concentrate their thoughts and all their efforts on proving themselves worthy of their re-defined civic identity (*politeuma*) in the new place in which they have been inscribed (*apegraphete*).^6^

In his *First Catechesis*, Chrysostom used mixed metaphors, first referring to the catechumens as soldiers of Christ and then comparing the act of baptism to a marriage ritual that is accompanied by the exchange of gifts between bride and groom. The abundant generosity and grace of God (his marriage-gift to the baptizand, as it were), he explains, is evident in the mere fact that “you have been deemed worthy to be inscribed as citizens (*politographêthênai*)”.^7^ He uses the same verb in his *Fourth Catechesis*, when he addresses the catechumens as soldiers of Christ who have just been inscribed as citizens in heaven.^8^ Later in the same work, he reminds his audience that “we have been inscribed in a different *politeia*, the Jerusalem above” so that they should show themselves in their deeds worthy of this distinction.^9^

*Politographesthai* is the technical term for the inscription of one’s name as a citizen in a specific city.^10^ Christian authors used it metaphorically to illustrate the significance of joining the Church through baptism, which is tantamount to inscription in the citizenship roll of the Church and the expectation of gaining full rights to the heavenly Jerusalem.

Basil of Caesarea (330–379) puts this especially eloquently in a homily on baptism, where he encourages the members of his audience to surrender themselves completely to God:

Change over to the side of the Lord. Give yourself the appellation [of being a Christian]. Enrol with the Church. The soldier is enrolled in lists, the athlete competes after registering himself, the member of a *deme* (*demotes*) who is enrolled as a citizen (*politographêtheis*) is counted among the members of a tribe (*phyletais*). In all these respects, you are answerable: as a soldier of Christ, as an athlete of piety, and as someone whose citizenship (*politeuma*) is in heaven. Have yourself inscribed, then, in that book, so that your name may be transferred above.^11^

Basil here seems to emphasise the contractual character of baptism, which goes hand in hand with his insistence on analogies with making a commitment in writing. Explaining that the Church is the foreshadowing of the *politeuma* in Heaven emphasises the communal aspect of the Christian life, shared by all those who observe the same conduct (*politeia*).

## Monastic contexts for *polis* language

Monks and holy men, in particular, were assumed to live out their heavenly citizenship to the fullest already during their earthly existence. The monastic enterprise anticipated the heavenly Jerusalem.^12^ The prologue to the *History of the Monks in Egypt* explains: “While living on earth in this manner they [the monks who follow Christ’s teaching] have their citizenship in heaven.”^13^ Next after baptism, a firm commitment to asceticism could thus secure citizenship rights in Heaven.

The *Life of Anthony* employs *polis* language on a large scale to celebrate its protagonist as the pioneering first hermit and founder of the monastic movement in Late Antique Egypt. It was composed shortly after Anthony’s death in 356, in the form of a letter sent to the brethren “abroad”, probably an ascetic community in the West. Its author Athanasius (296–373) endured several periods of exile for his stance against Arianism, which also coloured his attitude to the ruling emperors. As the first work of hagiography in Greek, the *Life of Anthony* enjoyed enormous popularity through the centuries and served as the blueprint for all later writing about monasticism and holy men. It has been subject to numerous scholarly studies, most recently by Jan Bremmer.^14^ Athanasius’s intentions in its composition have been scrutinised from many different angles, also in view of his other writings. They reveal a magnetic field where several tensions are held in balance. Athanasius casts his Anthony as a model of a miracle-working ascetic who draws large crowds of followers and yet lives in subordination to the clergy, as a hermit who choses life in the desert and yet remains in contact with urban settlements, as an unlearned simpleton who nevertheless is consulted by emperors and sought out as a disputation partner by erudite philosophers, as a solitary who pursues his asceticism alone, but who will receive his reward in the community of the Heavenly City.^15^

Thanks to the popularity of this *Vita* in subsequent generations and centuries, Anthony has traditionally been regarded as the founder of monasticism, and the eremitic life-style that he pursued as its primary and primeval organisational form from which communal monasticism later developed. This impression has been subject to serious revision thanks to a growing body of evidence from documentary papyri. Moreover, archaeological excavations in Egypt over recent decades have revealed a wide variety of monastic settlements for small and large groups. The old paradigm that pitches eremitic against cenobitic monasticism has now been replaced by a more nuanced appreciation of a large variety of social settings for the practice of the ascetic life, ranging from individuals to small family-like groups to moderately large associations centred around a spiritual father. Strictly organised large establishments that counted several dozen or more people, such as the Pachomian *koinonia* of monasteries for men and women, were only one option among many.^16^ Still, Anthony can justifiably be regarded as one of the great trail blazers for the monastic life at a distance from the cities. Or rather: his *Vita* established the discourse that pitches city against desert and asserts the primacy of eremitic monasticism, which would dominate monastic and hagiographical writing for subsequent centuries.

*polis* language plays a large role in this. In the words of Anthony’s biographer: “For there were not yet monastic dwellings in the hills and the desert had not yet been turned into a city of monks (*epolisthê monachôn*) who had departed from their previous lives and enrolled in the *politeia* of Heaven”.^17^ The metaphor of enlisting in the Heavenly City is malleable. As we have seen above, other fourth-century authors applied it to new members who joined the Church through baptism. Athanasius used it in a narrower sense for people who decided to abandon their social contexts and sought to pursue a different way of life, that of a monk.

*Politeia*, pious or ascetic conduct, is the key term of this text. Anthony has it,^18^ his followers wish to imitate it,^19^ the demons fear it. Athanasius is well informed about the Western addressees of the *Vita*: “For now there are monastic dwellings even in your region, and the designation of ‘monk’ has become a designation of citizenship (*kai to tôn monachôn onoma politeuetai*).”^20^ Anthony’s *politeia*, his conduct, is such that the demons are powerfully threatened by him and finally driven out altogether. When Satan finally admits defeat and retreats, he announces that he no longer has a place or a city (*polis*).^21^

Anthony’s death, at the age of 115 we are told, is depicted as a transition from a foreign city to his proper home city (*hôs apo allotrias eis idian apairôn polin*), another echo of Paul’s *politeuma* in Heaven.^22^

These reverberations of *polis*-thinking in monastic parlance are only present in the Greek text of the *Life of Anthony*, but are strikingly absent in the two Latin translations that were produced independently of each other within three decades. The *Vita Vetustissima* renders *politeia* and *politeuesthai* as *conversatio* and *conversationem habere*, while the translation by Evagrius (345–399) does not translate the relevant passages at all.^23^ *Conversatio* is also the Latin word used in the Vetus Latina and in Jerome’s Vulgata translations of the Letter to the Philippians 3:20. *Conversatio* vs *politeuma* – the different semantic fields covered by these different terms would invite further study. Could it be that, in the Latin West which was less densely urbanised than the Greek East, *polis* language had less resonance and relevance?

## Monastic jargon?

Athanasius may have composed the first work of hagiography, but Anthony was not the sole pioneer of the ascetic life, as he would have us believe. In Egypt from the late third century, disciples gathered around their spiritual leaders, or *abba*s, and formed monastic communities. Anthony himself is said to have been attended by two disciples at the time of his death. His younger contemporary Pachomius (292–348) attracted such a large following that, by the time of his death, there were seven monasteries for men and two for women that formed a loose association or *koinônia*. Pachomius introduced structured life in community as an organisational and economic necessity in order to facilitate a division of labour and tasks. He also established a set of rules in response to specific concerns as they arose. From then on, the eremitic and cenobitic monastic lifestyles coexisted, along with semi-eremitic monasticism in small family-like groups. The Systematic (i.e. thematically arranged) Collection of the *Sayings of the Desert Fathers* includes the following tell-tale passage:

A leader of a koinobion (communal monastery) once asked the blessed Cyril, Archbishop of Alexandria: “Who is better in conduct (*politeia*): we who have a crowd of brethren in our care and lead each one of them in a different way towards salvation, or those who live in the desert and only save themselves?”

The archbishop responded with Solomonic wisdom: “It is impossible to decide between Elijah and Moses. For both were pleasing to God.”^24^

The life of the great ascetics in Egypt attracted disciples and also pious visitors or pilgrims, who came in the hope of spiritual instruction and often stayed with an *abba* for a while. Their conversations, either in the form of aphorisms or short anecdotes, were first handed down orally from generation to generation, until they were written down, in varying combinations and sequences, circulating in writing far beyond the original circle of followers. The earliest written collections can be traced to fifth-century Palestine. Did these groups develop a particular way of expression? Did they use certain code words? Was there some kind of monastic jargon that the insiders shared?

In the 1950s, scholars around Christine Mohrmann in the Netherlands pursued the study of Late-Antique Latin in search of a specifically Christian way of expression in vocabulary and syntax. It is in this vein that A. Lorié observes with regard to the *Vita Antonii*: “‘Conversatio’ means 1. life or behaviour in its moral aspect; as the translation of πολιτεια it is a sort of technical term in early-Christian Latin; 2. it means social intercourse and in this it is of post-Classical origin.”^25^ Thanks to the recent work of Barbara Rosenwein on Merovingian Francia, it has become possible to identify “emotional communities” on the basis of their preferred use of certain words in a very specific sense and context.^26^

It is possible to make this case for the monastic environment with regard to *polis* language. While in classical Greek *politeia* has a variety of meanings, most prominently “body of citizens, government, constitution of a state”, in monastic contexts, it specifically means monastic lifestyle or ascetic conduct. In this sense, *politeia* occurs frequently in the *Sayings of the Desert Fathers*, 61 times, and the related verb, *politeuesthai*, “to lead an ascetic life”, occurs 13 times. Some examples may suffice: a desert father led a good ascetic life (*kalôs politeusamenos*); monks are encouraged to conduct themselves according to their monastic dress (*politeusômetha oun pros to schêma hêmôn*).^27^

It is in this sense that the title of the *Life of Anthony* should be read: while most ancient biographical narratives are called a *bios*, the *Life of Anthony* carries the title *bios kai politeia*, life and ascetic conduct. A very large number of Greek *lives* of saints – 78, to be exact, according to a search in the *Thesaurus Linguae Graecae* – use the same title.

There are even new idioms that are being created in the monastic context. One glimpse of this occurs in the *Sayings of the Desert Fathers*, when a hermit known for his strict lifestyle is described as “doing a lot of *politeiai* [pl!]” (*politeias poiôn pollas*).^28^ *Politeia* is here intended not as a general designation for an ascetic way of life, but for particular (albeit unspecified) ascetic practices, which are being performed with remarkable frequency.

Another use of insider jargon has gone unnoticed in previous scholarship. On three occasions in the *Life of Anthony*, the great hermit is said to “practice *politeia* against the devil” (*politeuesthai kat’ autou*), implying that ascetic practices can keep evil at bay.^29^ The usual and grammatically expected expression would be *politeuesthai kata ti* (in the accusative case): conducting oneself according to a particular custom or set of rules.^30^ The expression here, *politeuesthai kata tinos* (in the genitive case), by contrast, is highly unusual. The same expression was also used in the circle of Pachomius. According to his Greek *Vita*, probably composed around 390, Pachomius advised that the best way to protect oneself against demons was to be constantly watchful, to seal oneself with the name of Christ (by making the sign of the cross), and “to conduct oneself against them (*kat’ autôn politeuomenoi)*”, in other words: to engage in strict ascetic practices as a safeguard.^31^

Monks were distinguished by their asceticism to such an extent that, in the sixth century, Cyril of Scythopolis (525–558) in his biographies of the monks of the Judaean desert frequently employed the expression “monastic way of life” (*monachikê politeia*). That this way of life was exclusive to monks and distinguished them from outsiders is made clear by Cyril’s reference to: “*our* way of life” (*hê kat’ hêmas politeia*, emphasis mine).^32^In monastic jargon, then, *politeia* and *politeuesthai* has become shorthand for “doing the ascetic thing”.

Looking ahead to the seventh century, it is interesting to note that by then, the notion of a *politeia* that is specifically monastic had become such an integral part of Christian discourse that authors could suggest the inverse process: not only does monastic living resemble civic forms of engagement in the context of a *polis*, but cities can be conceived of as becoming “monasticised” through the adoption of liturgical practices originally associated with monks. This is expressed in Leontios of Neapolis’s (590–668) *Life of John the Almsgiver*, the pious patriarch of Alexandria who died in 619. John had constructed two churches when still a layman and later, during his patriarchate, installed a *tagma* (“order”) of monks in each of them. These monks were expected to observe vespers and night vigils in the church, in addition to optional liturgical rites in their cells. The hagiographer, who wrote several decades after the patriarch’s death, adds that these *tagmata* continue the practice of chanting hymns during vigils to the present day, so that “as a result, his [John’s] city conducts itself like a monastery” (*dikên monastêriou hê kat’ auton polis ek toutou politeueta*i).^33^

## *Politeuma* as a monastic term

What about the meaning of *politeuma* in monastic circles? In classical Greek, the word foregrounds the group identity of people who share the same way of life or code of conduct (*politeia*). Only in some rare instances are the two words used interchangeably.^34^ A prominent example is the *politeuma* of the Jews in Alexandria and other cities in Egypt, especially Herakleopolis.^35^ Eusebius of Caesarea (263–339), famous for his biography of the Emperor Constantine (r. 306–337) and his *Church History*, but an even more prolific author of biblical commentary and other theological texts, used the word often. His great agenda was to show that the Christians constitute the “true Israel” as the chosen people of God and heirs to God’s covenant with his people. He frequently preceded the word *politeuma* by the adjective *theosebes* (pious), when he referred to the Christians as a collective and intended the Church.^36^ By analogy, Eusebius identifies the Jews are designated as to *kata Môsea politeuma*. The same expression was frequently employed by Theodoret of Cyrrhus who refers to “the pious *politeuma*, that is the Church in the whole world (*oikoumenê*)”. His *Church History* presents a nuanced view of the possibilities to enact Christian civic identity, whether on earth or in Heaven.^37^ The designation as *politeuma* thus affirms the Christians as a recognisable group that is distinguished by worshipping God with the proper reverence.

In the monastic context, by contrast, *politeuma* primarily refers not to a group with features shared in common, but to the ascetic pursuits of the individual. In this sense, it is used almost interchangeably with *politeia*. Thus, Apa Kopres deflected praise for his asceticism by insisting that his way of life (*politeuma*) was not worthy of admiration compared with the conduct (*politeia*) of the fathers that he was striving to imitate.^38^

There is a striking absence of *polis*-language to express the communal aspects of monastic living. In search of the use of *politeuma* for the communal aspects of monasticism, we might turn to Basil of Caesarea, the first author to address the spiritual benefits of living in community, but without any results. In the set of rules that were assembled in his *Asketikon* that would remain influential in subsequent centuries, he did not use the word *politeuma* to refer to the community of monks, but *koinônia*.^39^ The same term was common among the confederation of Pachomian monasteries.^40^

Another context where we might expect concepts that relate to the social, communal aspect of the monastic life, analogous to that of a well-ordered city, is “the life of angels” (*angelikos bios*), a term often used for monasticism. Angels, after all, appear in groups, in hosts, (military) bands or as choirs (*chôros*). But this line of inquiry also leads to a dead end. As Karl Suso Frank has shown, Christian authors of Late Antiquity appreciated angel-like qualities not in the enactment of asceticism in community, but in the individual achievement of *askêsis*. The angelic life is attained by transforming one’s body through ascetic rigours, thus transcending one’s physical existence in anticipation of the Resurrection.^41^ When monastic authors speak of the life of angels with reference to the monastic enterprise, the emphasis is on the individual who is seeking to emulate the disembodied state of angelic existence: the absence of physical needs and desires, especially carnal appetites for food and sex. Only in this context, the dwelling place of the angels also comes into play, as they are imagined living close to God, in Heaven.^42^

This then, is a further significant observation: monastic authors do not employ *polis* language to elucidate the social aspects of monastic living. Their model for social organisation and interaction was not the *polis*, but the family, with spiritual fathers having spiritual sons and daughters, who are spiritual brothers and sisters.^43^

## Conclusion

This cursory sweep through Late-Antique Greek texts for the use of key terms of *polis* language in Greek monastic literature can serve as a supplement and corrective to previous observations. *Polis* language takes on a different tint in the context of proving one’s Christian dedication through the pursuit of the ascetic life as a monk, instead of joining the Church through baptism. The communal aspect of *polis* language that had been prominent in the ancient authors is no longer present. In the early centuries of Christian Byzantium, *politeia* becomes the standard expression in monastic and hagiographical writing for individual (not communal) conduct, and specifically for ascetic living. In this context, *polis* language develops its own dynamics, sometimes even as a jargon shared by insiders. This is a gradual process over time.^44^ Although the single steps along the way are not visible to us because of the lack of continuous evidence, the contours are clear. In parallel with the historic developments of the disappearance of large cities and the ruralisation of settlements in medieval Byzantium, the ancient *polis* as a self-governed community of citizens is becoming ever more distant, both in reality and as a concept. The *polis* of reference is no longer a city-state on earth, but a beautiful urban space in Heaven. Still, through adopting the *politeia* of ascetic living, its foreshadowing can be experienced here on earth.

## ORCID

*Claudia Rapp* http://orcid.org/0000-0001-9606-6888

## Footnotes

2 Claudia Rapp, “The Christianization of the Idea of the *Polis* in Early Byzantium”, in *Proceedings of the International Congress of Byzantine Studies, Sofia 2011*, volume I: *Plenary Papers*, ed. Ilija Iliev (Sofia: Bulgarian Historical Heritage Foundation, 2011), pp. 263–84; *eadem*, “City and Citizenship as Christian Concepts of Community in Late Antiquity”, in *The City in the Classical and Post-Classical World. Changing Contexts of Power and Identity*, ed. Claudia Rapp and Harold A. Drake (Cambridge: Cambridge University Press, 2014), pp. 153–66.

3 This phenomenon is now being studied at Cambridge, from a broader geographical perspective that also includes the Islamic world, in a project led by Andrew Wallace-Hadrill, “The Impact of the Ancient City” (https://www.classics.cam.ac.uk/research/projects/the-impact-of-the-ancient-city-1). The ideas in the current article were first presented at a workshop organised by this project in October 2018. I am grateful to the organisers and participants for this opportunity and for their valuable suggestions. There is an extensive and growing body of scholarship on the fate of cities in Late Antiquity. See for example J.H.W.G Liebeschuetz, *Decline and Fall of the Roman City* (Oxford: Oxford University Press, 2001); *Recent Research in Late Antique Urbanism*, ed. Luke Lavan (Portsmouth, RI: Journal of Roman Archaeology Supplementary Series 42, 2001); *New Cities in Late Antiquity: Documents and Archaeology*, ed. Efthymios Rizos (Turnhout: Brepols, 2017).

4 See Michael Hollerich, *Eusebius of Caesarea’s* Commentary on Isaiah (Oxford: Oxford University Press, 1999), pp. 103–30, for detailed studies of the use of relevant terminology by different authors.

5 For rich material and helpful analysis, see Helen Saradi, “The *Kallos* of the Byzantine City: The Development of a Rhetorical Topos and Historical Reality“, *Gesta* 34/1 (1995): 37–56.

6 John Chrysostom, *Catechesis* VII 12, in *Huit catéchèses inédits*, ed. Antoine Wenger [Sources Chrétiennes, volume L] (Paris : Éditions du Cerf, 2005), p. 235, ll. 9–12.

7 John Chrysostom, *Catechesis* I 18, in *ibid.*, p. 118, ll. 9–10.

8 John Chrysostom, *Catechesis* IV 6, in *ibid.*, p. 185, ll. 11–12.

9 John Chrysostom, *Catechesis* IV 29, in *ibid.*, p. 197, ll. 4–5. For inscription in the citizen list of the Heavenly Jerusalem, see also John Chrysostom, *In sanctum pascha* V, Patrologia Graeca, volume LII, col. 771.

10 See, for example, Diogenes Laertius, *Vitae philosophorum* V 84, ed. Miroslav Marcovich, volumes I–III (Stuttgart and Leipzig: Teubner, 1999), I: 367, l. 11.

11 Basil of Caesarea, *Homilia XIII exhortatoria ad sanctum baptisma* 7, Patrologia Graeca, volume XXXI, col. 440 A.

12 For an introduction to early monasticism and the texts it generated, see William Harmless, *Desert Christians: An Introduction to the Literature of Early Monasticism* (New York and Oxford: Oxford University Press, 2004). For a discussion of the concepts of desert and city among Christian and, especially, monastic authors, see Claudia Rapp, *Holy Bishops in Late Antiquity: The Nature of Christian Leadership in an Age of Transition* (Berkeley etc.: California University Press, 2005), pp. 111–14; and in greater detail *eadem*, “Desert, City and Countryside in the Early Christian Imagination”, in, *The Encroaching Desert: Egyptian Hagiography and the Medieval West*, ed. Jitse Dijkstra and Mathilde van Dijk [Dutch Archive of Church History=Church History and Religious Culture, volume CLLLVI] (Leiden: Brill, 2006), pp. 93–112.

13 Prologue 5, in *Historia monachorum in Aegypto*, ed. A.-J. Festugière (Brussels: Société des Bollandistes, 1971), p. 7, ll. 35–6. Unless otherwise stated, all translations are mine.

14 Jan N. Bremmer, “Athanasius’ *Life of Antony*: Marginality, Spatiality and Mediality”, in *Marginality, Media, and Mutations of Religious Authority in the History of Christianity*, ed. Laura Feldt and Jan N. Bremmer (Leuven: Peeters, 2019), pp. 23–46, with further bibliography. See also Ewa Wipszycka, “*Vita Antonii* [The Life of Anthony]”, in*The Second Gift of the Nile: Monks and Monasteries in Egypt* [Journal of Juristic Papyrology, Suppl., volume XXXIII] (Warsaw: University of Warsaw, 2018), pp. 27–107.

15 Susan Cartwright, “Athanasius’ ‘*Vita Antonii*’ as Political Theology: The Call of Heavenly Citizenship“, *Journal of Ecclesiastical History* 667/2 (2016): 241–64.

16 A first beginning were the seminal studies by James Goehring, *Ascetics, Society and the Desert: Studies in Early Egyptian Monasticism* (Harrisburg, PA: Trinity Press, 1999); see most recently Darlene Brooks Hedstrom, *The Monastic Landscape of Late Antique Egypt: An Archaeological Reconstruction* (Cambridge: Cambridge University Press, 2017); Ewa Wipszycka, “The Size of Monastic Communities”, in *The Second Gift of the Nile: Monks and Monasteries in Egypt* [Journal of Juristic Papyrology, Suppl. Volume XXXIII] (Warsaw: University of Warsaw, 2018), pp. 371–99). For an overview of the history of scholarship, see now also Albrecht Diem and Claudia Rapp, “The Monastic Laboratory: Perspectives of Research in Late Antique and Early Medieval Monasticism”, in *The New Cambridge History of Monasticism*, ed. Isabelle Cochelin and Alison Beach (Cambridge: Cambridge University Press, 2019), pp. 19–39.

17 *Life of Anthony* 14.7, in Athanase d’Alexandrie, *Vie d’Antoine*, ed. Gerard J.M. Bartelink [Sources Chrétiennes, volume CD] (Paris: Éditions du Cerf, 2004).

18 *Life of Anthony*, Proem. 2.

19 *Life of Anthony* 46.7.

20 *Life of Anthony*, Proem 1.

21 *Life of Anthony* 41.4.

22 *Life of Anthony* 89.4.

23 The relevant passages are: *Life of Anthony*, Proem. 2; 14.7; 24.6; 25.5; 28.5; 46.7. For the two Latin translations, see *Vitae Antonii versiones latinae*, ed. Pascal Bertrand and Lois Gandt [Corpus Christianorum, ser. lat., volume CLXX] (Turnhout: Brepols, 2018).

24 *Apophthegmata Patrum (collectio systematica)* 10.178, in Les apophtegmes des pères [ch*s* 10–16], ed. Jean-Claude Guy [Sources Chrétiennes, volume XDLXXIV] (Paris: Éditions du Cerf, 2003), p. 128.

25 Ludovicus Th. A. Lorié, *Spiritual Terminology in the Latin Translations of the* Vita Antonii (Nijmegen: Dekker & van de Vegt, 1955), pp. 82–5, esp. 82.

26 Barbara Rosenwein, *Emotional Communities in the Early Middle Ages* (Ithaca, NY: Cornell University Press, 2006).

27 *Apophthegmata Patrum (collectio systematica)* 7.62, in *Les apophtegmes des pères [chs 1–9],* ed. Jean-Claude Guy, [Sources Chrétiennes, volume CCCLXXXVII] (Paris: Éditions du Cerf, 1993), p. 394; *Apophthegmata (collectio anonyma)* (e cod. Coislin. 126), ch. 55, ed. F. Nau, "Histoires des solitaires égyptiens", *Revue de l'Orient Chrétien* 17–18 (1912–1913): 12:48–68, 171–181, 393–404, p. 180.

28 *Apophthegmata Patrum (collectio systematica)* 13.9, in Guy, *Les apophtegmes des pères [chs 10–16],* 236.

29 *Life of Anthony* 24.6, 25.5, 28.5.

30 For example Thucydides, *Peloponnesian War* 3.62.3, in *Thucydidis historiae*, ed. Henry Jones and John Powell, volume I (Oxford: Clarendon Press 1942, repr. 1970); Polybius, *Histories* 4.76 (*kata nomous*), in *Polybii historiae*, ed. T. Büttner-Wobst, volumes I–IV (Leipzig: Teubner: 1:1905, repr. 1962; 2:1889, repr. 1965; 3:1893, repr. 1965; 4:1904, repr. 1967); Clemens of Alexandria, *Paedagogus* 3.11.67 (*kata Theon*), ed. Marguerite Harl, Henri-Irénée Marrou, Claude Matray and Claude Mondésert, *Clément d'Alexandrie, Le pedagogue*, volume III [Sources Chrétiennes, volume CLVIII] (Paris: Éditions du Cerf, 1970), p. 134, l. 1; Origenes, *In Jeremiam homiliae* 9.3 (*kata Christon*), in *Origène. Homélies sur Jérémie*, ed. Pierre Nautin, volume I [Sources Chrétiennes, volume CCXXXII] (Paris: Éditions du Cerf, 1976), p. 386, ll. 32–3.

31 *Vita prima of Pachomius*, ch. 73, in *Sancti Pachomii Vitae graecae*, ed. Francois Halkin [Subsidia Hagiographica, volume XIX] (Brussels: Societé des Bollandistes, 1932), p. 49. Armand Veilleux, *Pachomian Koinonia*, volume I: *The Life of Saint Pachomius and his Disciples* [Cistercian Studies, volume XLV] (Kalamazoo, MI: Cistercian Studies Press, 1980), p. 348, translates “if you practise *ascesis* against them”. See also David Brakke, *Demons and the Making of the Monk: Spiritual Combat in Early Christianity* (Cambridge, MA: Harvard University Press, 2006), p. 92.

32 Cyril of Scythopolis, *Life of Euthymios*, in *Kyrillos von Skythopolis*, ed. E. Schwartz, [Texte und Untersuchungen, volume XLIX.2] (Leipzig: Hinrichs, 1939), pp. 3–85, esp. 50, l. 11; *Life of Sabas*, in *ibid.*, pp. 85–200, esp. 106, l. 5; *Life of John the Hesychast*, in *ibid.*, pp. 201–22, esp. 222, l. 1.

33 Leontios of Neapolis, *Life of John the Almsgiver* 48, in *Léontios de Néapolis, Vie de Syméon le Fou et Vie de Jean de Chypre*, ed. André-Jean Festugière and Lennart Rydén, (Paris: Paul Geuthner, 1974), p. 398, ll. 20–1.

34 Tatian, *Oratio ad Graecos* 19, 2, in *Die ältesten Apologeten*, ed. Edgar J. Goodspeed, (Göttingen: Vandenhoeck & Ruprecht, 1915), p. 286, ll. 5–6: “The world (*kosmos*) is created good, but the way of life (*politeuma*) in it is bad.”

35 See for example Thomas Kruse, “Zwischen Integration, Assimilation und Selbtsbehauptung: Das Politeuma der Juden von Herakleopolis in Mittelägypten“, in *Das Eigene und das Fremde*, ed. Andreas Pülz and Elisabeth Trinkl (Vienna: Verlag der Österreichischen Akademie der Wissenschaften, 2015).

36 Eusebius of Caesarea, *Commentaria in Psalmos*, Patrologia Graeca, volume XXIII, col. 441 B.

37 One example of many: Theodoret of Cyrrhus, *Interpretatio in Psalmos*, Patrologia Graeca, volume LXXX, col. 1216 B–C. See also the excellent study by Andreas Westergren, “Sketching the Invisible: Patterns of Church and City in Theodoret of Cyrrhus’ *Philotheos Historia*”, PhD Thesis, Lund University, 2012.

38 *Historia monachorum* 10 (Kopres), 76, ll. 11–14.

39 See for example, Basil of Caesarea, *Asceticon magnum sive Quaestiones (Regulae fusius tractatae)* 7, Patrologia Graeca, volumen XXXI, col. 932A.

40 Adalbert de Vogüé, “Foreword“, in *Pachomian Koinonia* I, trans. Armand Veilleux [Cistercian Studies, volumen XLV] (Kalamazoo, MI, 1980), pp. Vii–xxiii.

41 Karl Suso Frank, *Angelikos Bios: Begriffsanalytische und begriffsgeschichtliche Untersuchung zum* ‘*engelgleichen Leben’ im frühen Mönchtum* (Münster: Aschendorff, 1964), emphasises only the individual virtues associated with the angelic life.

42 See, for example, Evagrius Scholasticus, *Historia ecclesiastica*, in *The Ecclesiastical History of Evagrius with the Scholia*, ed. J. Bidez and J. Parmentier (London: Methuen, 1898), p. 21, l. 1: through his extreme asceticism, Symeon the Stylite was regarded as “an angel on earth, and a citizen of the Jerusalem above in the flesh” (*ho epi gês aggelos, ho en sarki tês anô Ierousalêm politês*).

43 The remark by Bernhard Kötting (“Genossenschaft. Christlich”, in *Reallexikon für Antike und Christentum*, volume X [Stuttgart: Hiersemann, 1978], cols 142–55, esp. 147) that Christians do not conceptualise their communities as analogous to *collegia*, but as analogous to the *polis*, thus stands in need of revision and expansion. For family concepts among early monks, see the relevant studies by Ville Vuolanto, *Children and Asceticism in Late Antiquity: Continuity, Family Dynamics and the Rise of Christianity* (Farnham: Ashgate, 2015).

44 In her perceptive study of the rhetorical form and literary and pedagogical function of the *Sayings of the Desert Fathers*, Lilian Larsen thus observes that monks should be seen as “adeptly appropriating the rhetorical and pedagogical tools of the classical world in refashioning the former *ecclesia* of the Greek city-state into a distinctive *ecclesia* of Christian people“ with the “humanistic formation of the ideal citizen“ as its goal. Lilian Larsen, “The *Apophthegmata Patrum* and the Classical Rhetorical Tradition”, in *Papers Presented at the Fourth International Conference on Patristic Studies held in Oxford 2003: Historica, Biblica Ascetica et Hagiographica*, ed. Frances Young, Mark Edwards and Paul Parvis [Studia Patristica, volume XXXIX] (Leuven: Peeters, 2006), pp. 409–415, esp. 414.

